# 2839. Cost Burden of Patients with Oral Antibiotic Treatment Failure for Uncomplicated Urinary Tract Infection in the United States

**DOI:** 10.1093/ofid/ofad500.2449

**Published:** 2023-11-27

**Authors:** Meg Franklin, Maia R Emden, Sharon Kautz, Anh Thy H Nguyen, Naomi C Sacks, Shinyoung Ju, Fanny S Mitrani-Gold, Ashish V Joshi, Madison T Preib

**Affiliations:** PRECISIONheor, Boston, MA, USA; Franklin Pharmaceutical Consulting, Cary, NC, USA, Boston, Massachusetts; PRECISIONheor, Boston, Massachusetts; PRECISIONheor, Boston, MA, USA, Boston, Massachusetts; PRECISIONheor, Boston, MA, USA, Boston, Massachusetts; PRECISIONheor, Boston, MA, USA, Boston, Massachusetts; GSK, Brentford, England, United Kingdom; GlaxoSmithKline plc., Collegeville, Pennsylvania; GlaxoSmithKline plc., Collegeville, Pennsylvania; GSK, Brentford, England, United Kingdom

## Abstract

**Background:**

Urinary tract infections (UTIs) are the most common bacterial infections in women. Antibiotics (ABX) are available to treat uncomplicated UTI (uUTI), but 10–30% of patients experience treatment failure (TF). We assessed uUTI-related healthcare costs among uUTI patients with/without oral ABX TF in the United States (US).

**Methods:**

This retrospective cohort study used Optum’s de-identified Clinformatics Data Mart Database from October 1, 2015 to September 30, 2021 (**Figure 1**). Eligible patients were female, ≥ 18 years, with a uUTI diagnosis identified in an outpatient setting, ≥ 1 oral ABX prescription within ± 5 days of diagnosis, and ≥ 1 year pre- and post-index follow-up. ABX prescription claim was designated index date.

uUTI-related costs were compared for patients with and without TF. TF was defined as any of the following during the initial uUTI episode (≤ 28 days post-index): ≥ 1 additional oral ABX prescription(s); intravenous ABX; or a primary diagnosis of UTI in an acute care setting (emergency room or inpatient). uUTI-related costs (US$) were assessed during the follow-up period.

**Results:**

In total, 238,335 patients were identified, of whom 29,333 (12.3%) had evidence of TF. Total uUTI-related costs were higher for patients with TF vs those without (mean [standard deviation, SD] $1133 [$5023] vs $276 [$1582]; p < 0.0001; [**Figure 2**]).

uUTI-related outpatient costs were higher for patients with vs without TF (mean [SD] $808 [$3368] vs $238 [$838]; p < 0.0001). Patients with TF had higher outpatient costs across all categories, with outpatient hospital visits contributing 45.6% of costs (**Figure 3**). By contrast, outpatient hospital visits accounted for < 20% of all outpatient costs for patients without TF; in these patients, office costs were the greatest contributor (31.9%).

uUTI-related inpatient costs were higher for patients with TF vs those without (mean [SD] $297 [$3562] vs $25 [$1326]; p < 0.0001). Mean (SD) pharmacy costs for patients with vs without TF were $28 ($26) and $13 ($16), respectively (p < 0.0001).

**Figure 1.**
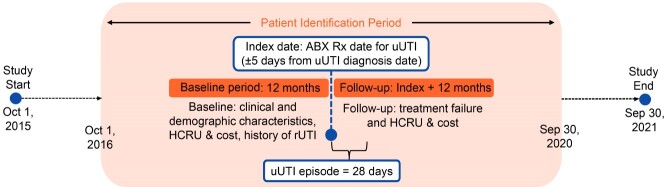
Study design ABX, antibiotic; HCRU, healthcare resource use; rUTI, recurrent urinary tract infection; uUTI, uncomplicated urinary tract infection; Rx, prescription.

**Conclusion:**

Patients with ABX TF for uUTI had significantly higher costs (inpatient, outpatient, and pharmacy) than patients without TF. This finding highlights the importance of appropriate empiric ABX selection to reduce the likelihood of TF in uUTI and its economic impact.

**Figure 2.**
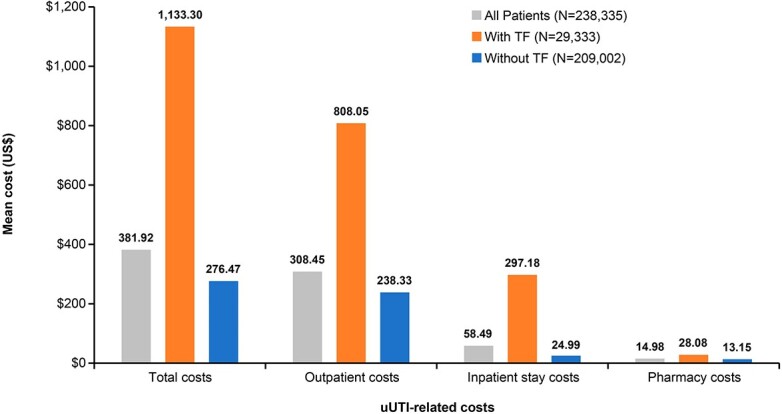
Mean uUTI-related costs during the follow-up period. All differences (patients with versus without treatment failure) were significant (p<0.0001). Outpatient costs = emergency room costs, outpatient hospital visits costs, office visits costs, lab and radiology tests costs, home health costs, durable medical equipment costs, and other outpatient costs. TF, treatment failure; uUTI, uncomplicated urinary tract infection.

**Figure 3.**
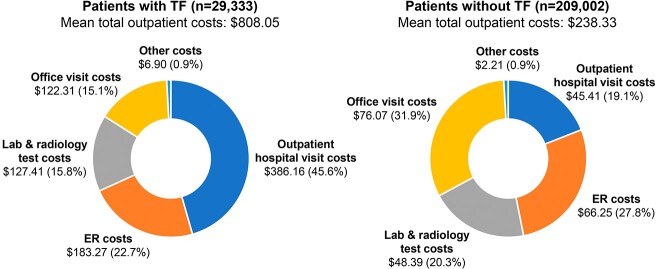
Distribution of uUTI-related outpatient costs in patients with versus without TF. Costs are in USD. Other costs = home health costs, durable medical equipment costs, and other outpatient costs. ER, emergency room; TF, treatment failure; USD, united states dollars; uUTI, uncomplicated urinary tract infection.

**Disclosures:**

**Meg Franklin, PharmaD, PhD**, Franklin Pharmaceutical Consulting: Owner and President|Franklin Pharmaceutical Consulting: Ownership Interest|GSK: Grant/Research Support|PRECISIONheor: Contractor **Maia R. Emden, BA**, GSK: Grant/Research Support|PRECISIONheor: Employee **Sharon Kautz, BS**, GSK: Grant/Research Support|PRECISIONheor: Employee **Anh Thy H. Nguyen, MSPH**, GSK: Grant/Research Support|PRECISIONheor: Employee **Naomi C. Sacks, PhD**, GSK: Grant/Research Support|PRECISIONheor: Employee **Shinyoung Ju, MS**, GSK: Employee|GSK: Stocks/Bonds **Fanny S. Mitrani-Gold, MPH**, GSK: Employee|GSK: Stocks/Bonds **Ashish V. Joshi, PhD**, GSK: Employee|GSK: Stocks/Bonds **Madison T. Preib, MPH**, GSK: Employee|GSK: Stocks/Bonds

